# Paricalcitol Improves Hypoxia-Induced and TGF-β1-Induced Injury in Kidney Pericytes

**DOI:** 10.3390/ijms22189751

**Published:** 2021-09-09

**Authors:** Jeong-Hoon Lim, Ju-Min Yook, Se-Hyun Oh, Soo-Jee Jeon, Hee Won Noh, Hee-Yeon Jung, Ji-Young Choi, Jang-Hee Cho, Chan-Duck Kim, Yong-Lim Kim, Sun-Hee Park

**Affiliations:** Department of Internal Medicine, School of Medicine, Kyungpook National University, Kyungpook National University Hospital, Daegu 41944, Korea; jh-lim@knu.ac.kr (J.-H.L.); jumin18@naver.com (J.-M.Y.); ttily@nate.com (S.-H.O.); hot716@naver.com (S.-J.J.); hwn1104@gmail.com (H.W.N.); hy-jung@knu.ac.kr (H.-Y.J.); jyss1002@hanmail.net (J.-Y.C.); jh-cho@knu.ac.kr (J.-H.C.); drcdkim@mail.knu.ac.kr (C.-D.K.); ylkim@knu.ac.kr (Y.-L.K.)

**Keywords:** hypoxia, paricalcitol, pericyte, pericyte–to–myofibroblast transition, TGF-β1, vitamin D agonist

## Abstract

Recently, the role of kidney pericytes in kidney fibrosis has been investigated. This study aims to evaluate the effect of paricalcitol on hypoxia-induced and TGF-β1-induced injury in kidney pericytes. The primary cultured pericytes were pretreated with paricalcitol (20 ng/mL) for 90 min before inducing injury, and then they were exposed to TGF-β1 (5 ng/mL) or hypoxia (1% O_2_ and 5% CO_2_). TGF-β1 increased α-SMA and other fibrosis markers but reduced PDGFRβ expression in pericytes, whereas paricalcitol reversed the changes. Paricalcitol inhibited the TGF-β1-induced cell migration of pericytes. Hypoxia increased TGF-β1, α-SMA and other fibrosis markers but reduced PDGFRβ expression in pericyte, whereas paricalcitol reversed them. Hypoxia activated the HIF-1α and downstream molecules including prolyl hydroxylase 3 and glucose transporter-1, whereas paricalcitol attenuated the activation of the HIF-1α-dependent molecules and TGF-β1/Smad signaling pathways in hypoxic pericytes. The gene silencing of HIF-1α vanished the hypoxia-induced TGF-β1, α-SMA upregulation, and PDGFRβ downregulation. The effect of paricalcitol on the HIF-1α-dependent changes of fibrosis markers was not significant after the gene silencing of HIF-1α. In addition, hypoxia aggravated the oxidative stress in pericytes, whereas paricalcitol reversed the oxidative stress by increasing the antioxidant enzymes in an HIF-1α-independent manner. In conclusion, paricalcitol improved the phenotype changes of pericyte to myofibroblast in TGF-β1-stimulated pericytes. In addition, paricalcitol improved the expression of fibrosis markers in hypoxia-exposed pericytes both in an HIF-1α-dependent and independent manner.

## 1. Introduction

Renal interstitial fibrosis is a common pathologic consequence of different kinds of kidney injuries, and transforming growth factor (TGF)-β1 is a main contributor to induce kidney fibrosis. Hypoxia is also known to be associated with the progressive loss of kidney function, while affecting various cell populations in the kidney. Various humoral factors from damaged tubular epithelial cells and infiltrating inflammatory cells trigger the transformation of interstitial resident cells such as pericytes into myofibroblasts. The genetic lineage tracing technique has shown that pericytes are the origin of myofibroblasts in kidney fibrosis [1]. Progressive tubulointerstitial fibrosis causes kidney hypoxia, which worsens fibrosis, thus forming a vicious cycle [2]. Hypoxia-exposed cells upregulate hypoxia-inducible factor (HIF)-1α, which is the main transcription factor needed to adapt to hypoxic conditions by regulating oxygen homeostasis and the cell metabolism. HIF-1α is known to play different roles in various kidney cells [3]. HIF-1α is associated with chronic kidney disease (CKD) progression and kidney fibrosis by activating the TGF-β1-dependent pathway and mediating the epithelial–to–mesenchymal transition [4].

Pericytes, which are branched mesenchymal cells surrounding the endothelial cells in the capillary bed and postcapillary venules, have hemostatic functions during the process of angiogenesis and are involved in vasomotor tone regulation. Moreover, pericytes have a key role in the process of initiation of and the progression of vascular or fibrogenic diseases. Accumulating evidence has revealed the function of kidney pericytes, such as regulating renal blood flow, microvessel stability, and endothelial survival as well as having an essential role in kidney injuries (e.g., acute kidney injury [AKI] and CKD) [2,5,6].

A previous study demonstrating that inflammatory stimulus induced the expression of CYP2R1 (vitamin D 25-hydroxylase) and vitamin D receptors (VDR) in brain pericytes [7] led us to the hypothesis that VDR activators may be associated with the mitigation of pericyte injury in the kidney.

Therefore, the aim of the present study is to evaluate the effects of paricalcitol on hypoxia-induced and TGF-β1-induced injury in kidney pericytes.

## 2. Results

### 2.1. Protective Effects of Paricalcitol on TGF-β1-Induced Pericyte–to–Myofibroblast Transition

**Figure 1:**
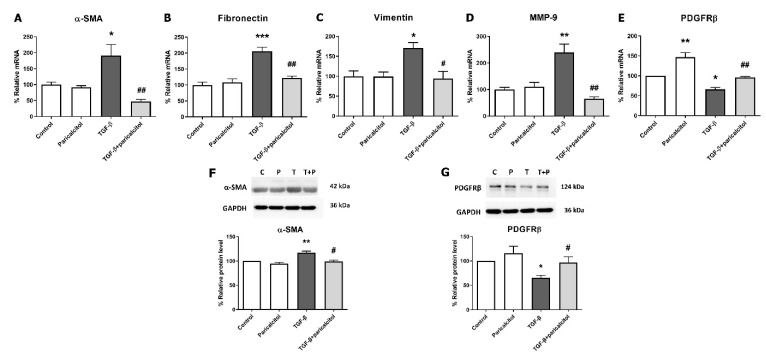
Effect of paricalcitol on TGF-β1-induced fibrosis. (**A**–**C**) TGF-β1 increased the mRNA expression of the profibrotic mesenchymal markers, α-SMA, fibronectin, and vimentin, and paricalcitol decreased the fibrotic markers in pericytes. (**D**) The MMP-9 mRNA expression was increased after TGF-β1 stimulation, and paricalcitol attenuated the MMP-9 expression. (**E**) The PDGFRβ mRNA (pericyte marker) expression was decreased after TGF-β1 treatment, and paricalcitol increased the PDGFRβ expression. (**F**,**G**) The protein expression of α-SMA was decreased and PDGFRβ was increased after TGF-β1 stimulation. Paricalcitol cotreatment reversed the changes of α-SMA and PDGFRβ protein levels. The data are presented as means ± standard errors. *n* = 4 per each group. * *p* < 0.05 vs. control; ** *p* < 0.01 vs. control; *** *p* < 0.001 vs. control; ^#^ *p* < 0.05 vs. TGF-β1 treatment group; ^##^ *p* < 0.01 vs. TGF-β1 treatment group. Abbreviations: α-SMA, alpha-smooth muscle actin; MMP-9, matrix metalloproteinase-9; PDGFRβ, platelet-derived growth factor receptor beta.

TGF-β1 stimulation increased mRNA expression of profibrotic markers such as alpha-smooth muscle actin (α-SMA), fibronectin, vimentin, and matrix metalloproteinase-9 (MMP-9) in a dose-dependent manner (Supplemental Figure S1). To evaluate the effect of paricalcitol on TGF-β1-induced fibrosis in pericytes, TGF-β1 exposure was performed after paricalcitol pretreatment. The mesenchymal markers were measured using realtime PCR; the α-SMA, fibronectin, and vimentin mRNA expressions were significantly increased after TGF-β1 stimulation (all *p* < 0.05) (Figure 1A–C). Paricalcitol cotreatment attenuated the upregulation of the mesenchymal markers, such as α-SMA, fibronectin, and vimentin expressions (all *p* < 0.05). The mRNA expression of MMP-9, which is involved in the degradation of the extracellular matrix (ECM), was also increased, whereas the paricalcitol cotreatment downregulated the MMP-9 expression (*p* < 0.05) (Figure 1D). As a pericyte marker, platelet-derived growth factor receptor beta (PDGFRβ) mRNA level was decreased after TGF-β1 stimulation, and paricalcitol increased the PDGFRβ expression compared with both the control and TGF-β1-exposed groups (both *p* < 0.05) (Figure 1E). The protein expression of α-SMA was increased and PDGFRβ was decreased after TGF-β1 stimulation (both *p* < 0.05) (Figure 1F,1G), whereas paricalcitol cotreatment reversed the changes of both α-SMA and PDGFRβ protein levels (both *p* < 0.05). These results suggested that TGF-β1 exposure causes pericyte–to–myofibroblast transition (PMT) and that paricalcitol treatment improves PMT.

### 2.2. Paricalcitol Attenuated Smad2 Phosphorylation in TGF-β1-Induced Fibrosis in Pericytes

**Figure 2:**
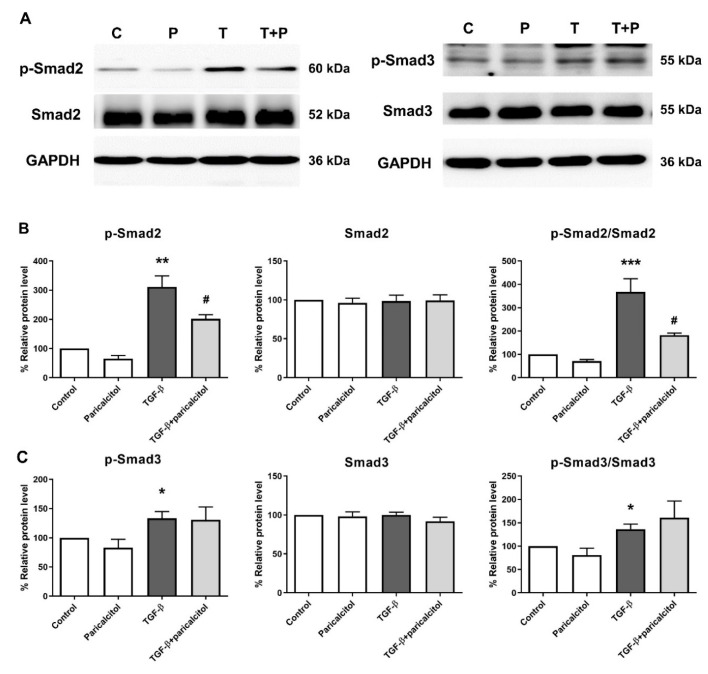
Expression of Smad2 and Smad3 in TGF-β1- exposed pericytes. (**A**,**B**) TGF-β1 significantly increased both the p-Smad2 level and the ratio of p-Smad2 to Smad2 in pericytes, and they were decreased after paricalcitol treatment. (**A**,**C**) Moreover, TGF-β1 significantly increased both the p-Smad3 level and the ratio of p-Smad3 to Smad3, and they were not altered after paricalcitol treatment. The data are presented as means ± standard errors. *n* = 4 per each group. * *p* < 0.05 vs. control; ** *p* < 0.01 vs. control; *** *p* < 0.001 vs. control; ^#^ *p* < 0.05 vs. TGF-β1 treatment group. Abbreviations: C, control group; P, paricalcitol treatment group; T, TGF-β1 treatment group; T + P, TGF-β1 and paricalcitol cotreatment group.

We investigated the mechanism of the effect of paricalcitol treatment on the Smad-dependent pathway in TGF-β1-induced PMT. The protein expressions of the Smad2 and Smad3 pathways were measured through western blot analysis (Figure 2). The level of p-Smad2 and the ratio of p-Smad2 to Smad2 were significantly increased after TGF-β1 exposure, and such increase was alleviated after par1icalcitol cotreatment (Figure 2B). The level of p-Smad3 and the ratio of p-Smad3 to Smad3 were also increased after TGF-β1 exposure (both *p* < 0.05), but the paricalcitol cotreatment did not change the levels of p-Smad3 and the ratio of p-Smad3 to Smad3 (both *p* > 0.05) (Figure 2C).

### 2.3. Paricalcitol Decreased TGF-β1-Induced Migration of Pericytes

**Figure 3:**
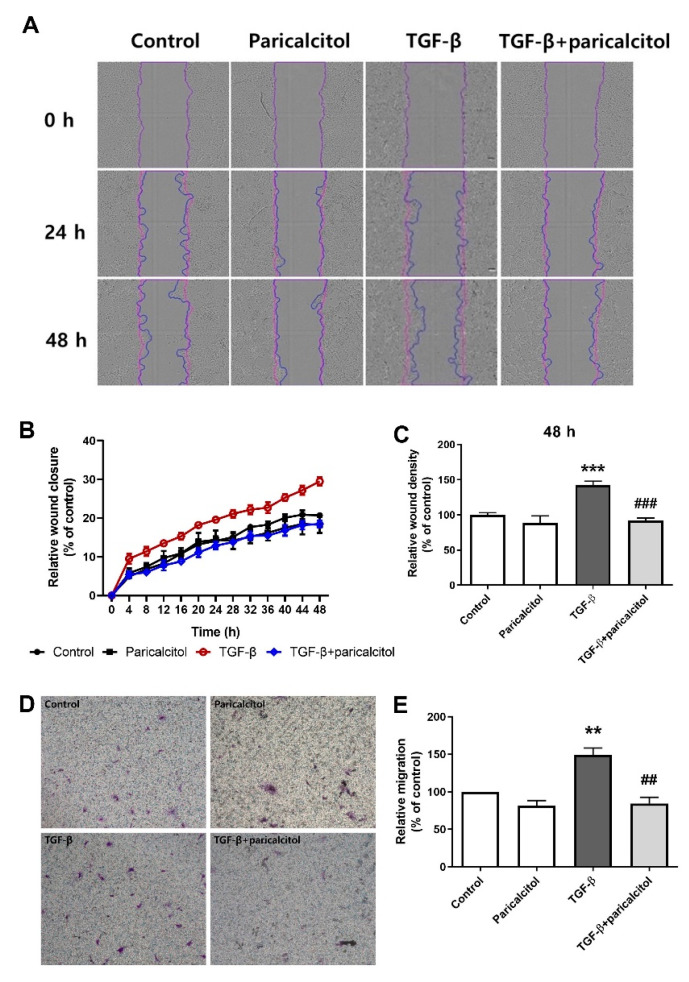
Effect of paricalcitol on TGF-β1-induced migration in pericytes in the scratch and transwell cell migration assays. (**A**) Light micrographs of scratched cell monolayers were taken at 0, 24, and 48 h with TGF-β1 and/or paricalcitol. (**B**) The relative scratch cell migration was measured over time using the Incucyte live imaging platform every 4 h up to 48 h after scratch cell migration. (**C**) Relative scratch cell migration at 48 h. *n* = 4 per each group. (**D**) The representative microscopic images of pericytes that had migrated (stained with 0.1% crystal violet; original magnification, ×100). (**E**) The relative migration was determined by comparing the number of migrated pericytes, and they were counted in 10 randomly selected fields in each group. *n* = 4 per each group. The data are presented as means ± standard errors. ** *p* < 0.01 vs. control; *** *p* < 0.001 vs. control; ^##^ *p* < 0.01 vs. TGF-β1 treatment group; ^###^ *p* < 0.001 vs. TGF-β1 treatment group.

To determine whether TGF-β1 induces pericyte migration and evaluate the role of paricalcitol in the migration, we examined the pericyte migration after TGF-β1 stimulation with or without paricalcitol cotreatment. TGF-β1 significantly accelerated the wound closure compared with the control pericytes in the scratch cell migration assay, whereas the cotreatment of TGF-β1 and paricalcitol delayed the scratch wound closure of pericytes (Figure 3A–C).

Furthermore, transwell migration assay was performed to confirm the effect of paricalcitol on pericyte migration through the membrane. TGF-β1 significantly increased the pericyte mobility, whereas the paricalcitol cotreatment decreased the TGF-β1-induced increase in migration (Figure 3D,E). Overall, paricalcitol treatment improved the migratory changes of pericyte after TGF-β1 exposure.

### 2.4. Protective Effect of Paricalcitol on Hypoxia-Induced Fibrosis in Pericytes

**Figure 4:**
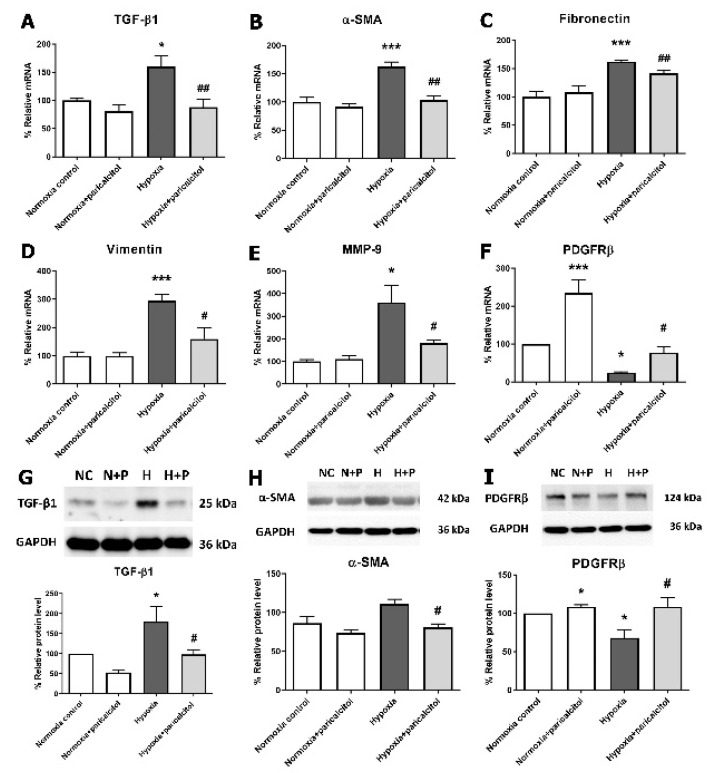
Effect of paricalcitol on TGF-β1 and fibrosis markers in hypoxic pericytes. (**A**) Hypoxia increased the TGF-β1 mRNA expression, whereas the paricalcitol treatment decreased the TGF-β1 mRNA level. (**B**–**D**) Hypoxia increased the mRNA expressions of profibrotic mesenchymal markers, α-SMA, fibronectin, and vimentin, whereas paricalcitol decreased the fibrotic markers in pericytes. (**E**) MMP-9 mRNA expression was increased under hypoxic condition, and paricalcitol attenuated the MMP-9 expression. (**F**) Hypoxia decreased the mRNA expression of PDGFRβ compared to normoxic control, whereas paricalcitol increased PDGFRβ expression compared to hypoxic pericyte. (**G**) Hypoxia increased the TGF-β1 protein level, whereas paricalcitol treatment decreased the TGF-β1 protein level. (**H**,**I**) Paricalcitol treatment reversed protein expression of α-SMA in hypoxic pericytes. The PDGFRβ protein level was decreased in hypoxia-exposed pericytes, and paricalcitol also reversed the PDGFRβ protein expression. The data are presented as means ± standard errors. *n* = 4 per each group. * *p* < 0.05 vs. normoxia control; *** *p* < 0.001 vs. normoxia control; ^#^ *p* < 0.05 vs. hypoxia group; ^##^ *p* < 0.01 vs. hypoxia group. Abbreviations: α-SMA, alpha-smooth muscle actin; MMP-9, matrix metalloproteinase-9; PDGFRβ, platelet-derived growth factor receptor beta.

Next, we investigated the changes of pericyte- and fibrosis-related markers in hypoxia-exposed pericytes. Both the TGF-β1 gene expression and protein levels were significantly increased under hypoxic condition compared with those under normoxic condition (both *p* < 0.05) (Figure 4A,G). Paricalcitol treatment in hypoxia-exposed pericytes reversed both the upregulated TGF-β1 gene and protein levels compared with no paricalcitol treatment (both *p* < 0.05). Mesenchymal markers, such as α-SMA, fibronectin, and vimentin, and MMP-9 showed an increased mRNA expression in hypoxic pericytes (all *p* < 0.05), whereas paricalcitol treatment reversed the upregulated α-SMA, fibronectin, vimentin, and MMP-9 mRNA expression (all *p* < 0.05) (Figure 4B–E). Paricalcitol treatment also reversed the protein expression of α-SMA in the hypoxic pericytes (*p* < 0.05) (Figure 4H). The mRNA and protein expressions of PDGFRβ were decreased in hypoxia-exposed pericytes, and paricalcitol reversed the PDGFRβ mRNA and protein expressions compared to both control and hypoxic pericytes (all *p* < 0.05) (Figure 4F,I). These results indicated that hypoxia induces PMT, which is associated with an increase in TGF-β1, whereas paricalcitol downregulates hypoxia-induced PMT.

### 2.5. Paricalcitol Attenuated Smad2 Phosphorylation in Hypoxia-Exposed Pericytes

**Figure 5:**
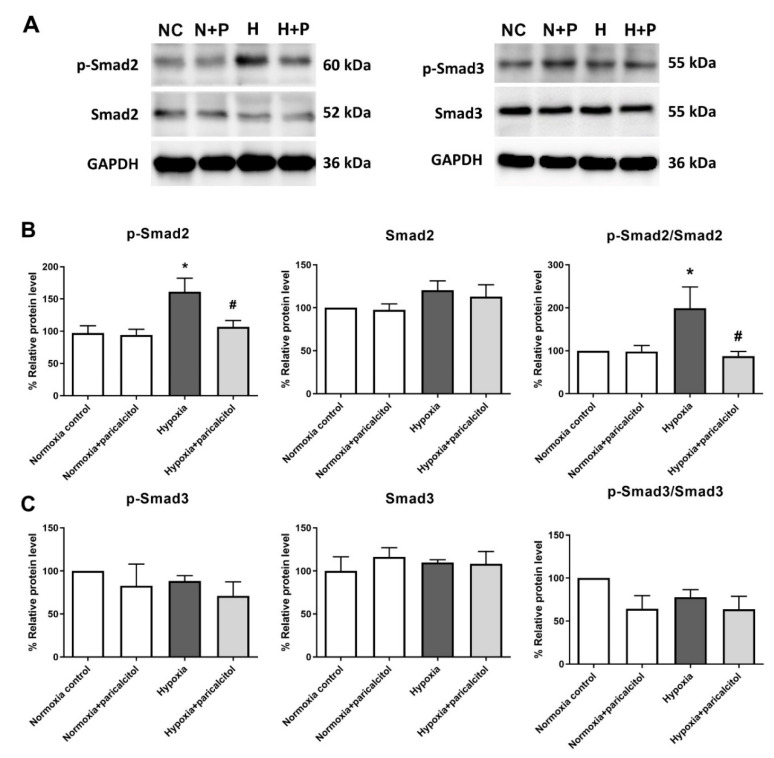
Expression of Smad2 and Smad3 in hypoxic pericytes. (**A**,**B**) Exposure to hypoxia significantly increased both the p-Smad2 level and the ratio of p-Smad2 to Smad2 in pericytes, and they were decreased after paricalcitol treatment. (**A**,**C**) Hypoxia did not alter both the p-Smad3 level and the ratio of p-Smad3 to Smad3, and they were not decreased after paricalcitol treatment. The data are presented as means ± standard errors. *n* = 4 per each group. * *p* < 0.05 vs. control; ^#^ *p* < 0.05 vs. hypoxia group. Abbreviations: NC, normoxia control group; N+P, normoxia and paricalcitol treatment group; H, hypoxia group; H+P, hypoxia and paricalcitol treatment group.

We examined the regulation of the Smad signaling pathway in pericytes during hypoxia via western blot analysis. The expression of p-Smad2 and the ratio of p-Smad2 to Smad2 were significantly upregulated in pericytes exposed to hypoxia, whereas paricalcitol reversed the upregulation of the Smad2 pathway in hypoxia-exposed pericytes (both *p* < 0.05) (Figure 5A,B). However, paricalcitol did not change the upregulated Smad3 signaling pathway under hypoxic condition (*p* > 0.05) (Figure 5A,C).

### 2.6. Paricalcitol Attenuated Hypoxia-Induced Pericyte Injury in an HIF-1α-Dependent Manner

**Figure 6:**
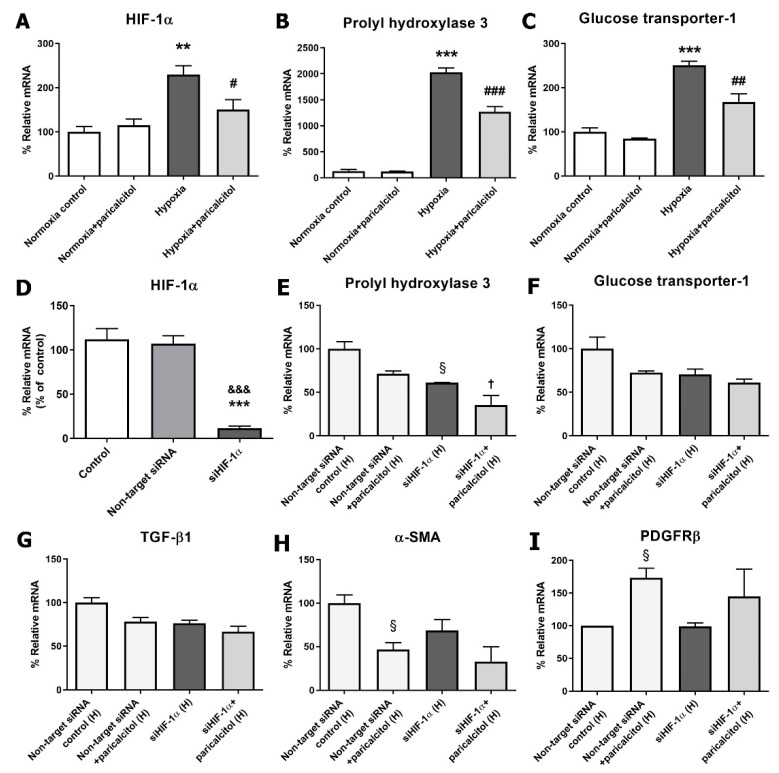
Effect of paricalcitol on hypoxia-induced HIF-1α activation and after the gene silencing of HIF-1α. (**A**–**C**) Paricalcitol attenuated the hypoxia-induced HIF-1α and HIF-1α-dependent gene (PHD3 and GLUT-1) expressions. (**D**) The transfection of pericytes with HIF-1α siRNA (50 μM, 6 hr) significantly inhibited the HIF-1α expression compared with the control and nontarget siRNA groups. (**E**–**G**) The paricalcitol effects on TGF-β1, PHD3, and GLUT-1 were diminished after the gene silencing of HIF-1α. (**H**,**I**) The paricalcitol effects on α-SMA and PDGFRβ were diminished after the gene silencing of HIF-1α. The data are presented as means ± standard errors. *n* = 4 per each group. ** *p* < 0.01 vs. normoxia control; *** *p* < 0.001 vs. normoxia control; ^#^ *p* < 0.05 vs. hypoxia group; ^##^ *p* < 0.01 vs. hypoxia group; ^###^ *p* < 0.001 vs. hypoxia group; ^&&&^ *p* < 0.001 vs. nontarget siRNA normoxia group; ^§^ *p* < 0.05 vs. nontarget siRNA hypoxia control; ^†^ *p* < 0.05 vs. siHIF-1α hypoxia group. Abbreviations: HIF, hypoxia-inducible factor; PHD3, prolyl hydroxylase 3; GLUT-1, glucose transporter-1; siRNA, small interfering RNA; H, hypoxia; α-SMA, alpha-smooth muscle actin; PDGFRβ, platelet-derived growth factor receptor beta.

Hypoxia induced the upregulation of the HIF-1α gene expression compared with normoxic pericytes, and paricalcitol cotreatment attenuated the HIF-1α expression (*p* < 0.05) (Figure 6A). HIF-1α-dependent gene expressions, such as prolyl hydroxylase 3 (PHD3) and glucose transporter-1 (GLUT-1), were also increased under hypoxic condition, but decreased after paricalcitol cotreatment (both *p* < 0.05) (Figure 6B,C). These results indicated that paricalcitol downregulates the activation of the HIF-1α-dependent pathway during hypoxia.

To investigate the role of HIF-1α in hypoxic injury in pericytes and the action site of paricalcitol, we evaluated the effect of the gene silencing of HIF-1α using small interfering (si)RNAs. Figure 6D shows the effective inhibition of HIF-1α by HIF-1α siRNA. The TGF-β1 did not increased by gene silencing of HIF-1α in hypoxia-exposed pericytes, and the paricalcitol treatment did not significantly decrease the TGF-β1 levels after the gene silencing of HIF-1α (Figure 6G). Under hypoxic condition, the HIF-1α-dependent gene expressions, such as PHD3 and GLUT-1, also did not increase after the gene silencing of HIF-1α. The paricalcitol treatment further decreased the mRNA expression of PHD3 (Figure 6E) but did not change the mRNA expression of GLUT-1 (Figure 6F). The mRNA expression of α-SMA and PDGFRβ were also not significantly changed by paricalcitol treatment after treatment with HIF-1α siRNA in hypoxic pericytes (Figure 6H,I).

### 2.7. Paricalcitol Attenuated Hypoxia-Induced Oxidative Stress in an HIF-1α-Independent Manner

**Figure 7:**
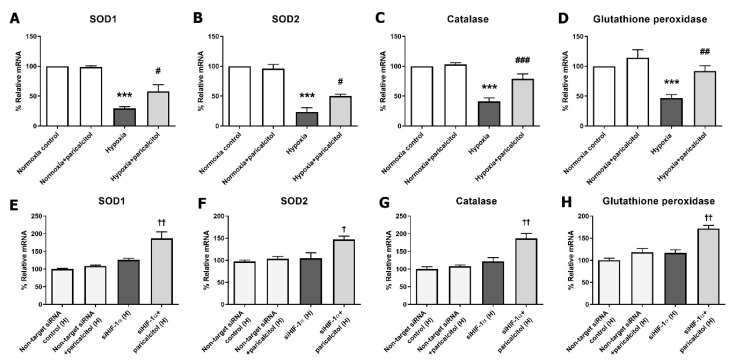
Effect of paricalcitol on antioxidant enzymes in hypoxic pericytes with or without the gene silencing of HIF-1α. (**A**–**D**) The oxidative stress-related antioxidant enzymes (SOD1, SOD2, catalase, and glutathione peroxidase) were significantly decreased in hypoxic pericytes, whereas the antioxidant enzymes were increased after paricalcitol treatment. (**E**–**H**) The antioxidant enzymes were significantly increased after paricalcitol treatment in hypoxic pericytes with the gene silencing of HIF-1α. The data are presented as means ± standard errors. *n* = 4 per each group. *** *p* < 0.001 vs. normoxia control; ^#^ *p* < 0.05 vs. hypoxia group; ^##^ *p* < 0.01 vs. hypoxia group; ^###^ *p* < 0.001 vs. hypoxia group; ^†^ *p* < 0.05 vs. siHIF-1α hypoxia group; ^††^ *p* < 0.01 vs. siHIF-1α hypoxia group. Abbreviations: HIF, hypoxia-inducible factor; SOD, superoxide dismutase; siRNA, small interfering RNA; H, hypoxia.

Figure 7 shows the effect of paricalcitol on oxidative stress injury in hypoxia-exposed pericytes. We measured the changes of the antioxidant enzymes, including superoxide dismutases (SODs), catalase, and glutathione peroxidase, in hypoxic pericytes with and without the gene silencing of HIF-1α. In the hypoxia-exposed pericytes, the antioxidant enzyme levels (SOD1, SOD2, catalase, and glutathione peroxidase) were decreased, but they increased after paricalcitol treatment (Figure 7A–D). After the gene silencing of HIF-1α in pericytes, the paricalcitol treatment upregulated the expression of the antioxidant enzymes (SOD1, SOD2, catalase, and glutathione peroxidase) under hypoxic condition (all *p* < 0.05) (Figure 7E–H). Furthermore, the results indicated that paricalcitol may also have antioxidant effects in hypoxic pericytes, and these effects are independent to the expression of HIF-1α.

## 3. Discussion

The present study demonstrated the protective effect of paricalcitol against hypoxia-induced and TGF-β1-induced injury in pericyte. A crosstalk can be observed between kidney fibrosis and tissue hypoxia, and they synergistically promote CKD progression [2]. Both TGF-β1 stimulation and exposure of hypoxia induce PMT, and paricalcitol treatment improves the fibrotic changes in injured pericytes. The antifibrotic effect of paricalcitol was associated with the HIF-1α-dependent Smad2 signaling pathway and its downregulation. Moreover, paricalcitol had antioxidant effects in hypoxia-exposed pericytes in an HIF-1α-independent manner, which may also inhibit PMT.

Paricalcitol, 19-nor-1,25-(OH)_2_-vitamin D_2_, is an active vitamin D analog and selectively activates VDR. It is mainly used to treat secondary hyperparathyroidism, and its additional antifibrotic and antioxidant effects have been proven in previous studies [8,9,10]. Although the nonselective vitamin D activators also showed similar beneficial effects, such as anti-inflammation and immunomodulation [11,12], they tend to increase the serum calcium and phosphate levels. Paricalcitol is a selective VDR agonist and has beneficial effects on calcium homeostasis without increasing the electrolyte levels. It has demonstrated many beneficial effects on kidney diseases in experimental studies, such as those on diabetic nephropathy, obstructive nephropathy, and ischemic kidney injury [13,14,15]. Pericytes are ubiquitously present in all vascularized tissues including the kidneys and have mediate basic functions related to the structural stabilization of the microvasculature, blood flow control, angiogenesis, and wound healing [5,6,16,17,18]. Quiescent pericytes are activated and altered by several factors, such as TGF-β1 and oxidative stress, which are upregulated in kidney injuries or hypoxic conditions [2,6,19]. Activated pericytes are detached from the peritubular capillaries and converted into myofibroblasts and consequently cause kidney fibrosis [2]. Furthermore, we have shown the protective effects of paricalcitol on hypoxia-induced and TGF-β1-induced fibrosis in pericytes, which might contribute to the inhibition of CKD progression.

Kidney fibrosis is a common pathologic feature of CKD regardless of its cause, and it is a hallmark of CKD that predicts long-term kidney function and progression to endstage kidney disease (ESKD) [2,20,21]. In the present study, TGF-β1 stimulation, hypoxia-induced PMT, and paricalcitol treatment attenuated the profibrotic process by inhibiting the TGF-β1/Smad signaling pathway. Kidney myofibroblasts, which are activated mesenchymal cells that express α-SMA, play an important role in kidney fibrosis through the generation and accumulation of the ECM in the tubulointerstitium which constitutes kidney scar [6]. Regarding the antifibrotic therapy for preventing CKD progression, researchers have investigated the main target cells and mediators that are associated with the proliferation of myofibroblasts [22,23,24]. Moreover, the exact origin of myofibroblasts is yet to be determined. They are known to originate from various cells, such as pericytes, resident fibroblasts, epithelial cells, endothelial cells, and stromal mesenchymal cells [25]. However, particularly in the kidneys, Humphreys et al. reported that most of these activated mesenchymal fibroblasts are attached to the microvasculature and originated from pericytes [19]. Interestingly, paricalcitol solely downregulated the Smad2 signaling pathway, but not the Smad3 pathway, in both hypoxia-induced and TGF-β1-induced injury of pericytes in this study. Smad2 and Smad3 share a 90% similarity in their amino acid sequences, but they have differential sensitivity in relaying TGF-β1 signaling [26]. Although the exact mechanism on how paricalcitol inhibits the Smad2 pathway cannot be explained in this study, the inhibition of the phosphorylation of Smad2 may be a major mechanism for the antifibrotic effect of paricalcitol in kidney pericytes.

Kidneys are physiologically hypoxic organs, despite having a large amount blood supply (20% of the cardiac output in humans), because of the arterial–venous oxygen shunt [27,28,29]. Moreover, the oxygen supply to the kidney cortex can change dramatically depending on the status of the renal perfusion [30]. Since these bring inefficient utilization of oxygen and variability in the intrarenal oxygen levels, the kidneys tend to be vulnerable to hypoxic injuries [30]. In the acute phase of AKI, renal tubular epithelial cells and vascular endothelial cells are damaged and activated. This results in the upregulation of various cytokines and chemokines, consequently the inflammatory cascades are initiated, and injury aggravates [31,32,33]. In the chronic phase of AKI, AKI aggravates kidney hypoxia by inducing tubulointerstitial fibrosis, because the excessive deposition of ECM increases the distance between the peritubular capillaries and tubular epithelial cells, resulting in a decrease in oxygen diffusion efficiency [27]. Capillary rarefaction via pericyte detachment with a decreased expression of vascular factors is also an important cause of kidney hypoxia. Hypoxia activates the HIF-1α signaling pathway, which leads to the integration of multiple signaling networks, such as TGF-β/Smad, Notch, and NF-κB pathways, to induce kidney fibrosis [3]. This vicious cycle between kidney fibrosis and hypoxia contributes to the progression of CKD. Thus, these are the main therapeutic targets in kidney injury.

To clearly evaluate the hypoxic changes of pericytes and the protective effect of paricalcitol in a similar environment to kidney injury, the pericytes were exposed to hypoxic conditions. Exposure to hypoxia increased the HIF-1α and HIF-1α-dependent gene expressions. HIF-1α causes kidney fibrosis and reduces oxygen utilization by inhibiting the metabolic pathways and intracellular organelles that consume oxygen [34,35,36]. Paricalcitol showed an antifibrotic effect by downregulating HIF-1α, subsequent HIF-1α-dependent genes, and the TGF-β1 expressions in hypoxia-exposed pericytes. The results of the gene silencing of HIF-1α also support that HIF-1α would be a main therapeutic target of paricalcitol in hypoxic pericyte injury. Meanwhile, the PHD3 expression decreased after paricalcitol treatment even after the silencing of HIF-1α, although the degree of decrease was weakened. This may be related to the fact that PHD3 not only is an oxygen sensor but also induces apoptosis through the HIF-1α-independent mechanism [37]. However, further studies on the antiapoptotic effect of paricalcitol in pericytes are needed. Furthermore, paricalcitol has shown to reduce oxidative stress injury in hypoxia-exposed pericytes in an HIF-1α-independent manner. Oxidative stress is a well-known factor that contributes to kidney fibrosis and inflammation [38]. Overall, renal hypoxia activates PMT to develop CKD, whereas paricalcitol can inactivate fibrosis in an HIF-1α-dependent and HIF-1α-independent manner.

Recently, many studies have reported the importance of hypoxia in AKI-to-CKD transition [39,40]. Fibrosis is an essential protective response to tissue injury itself, but chronic excessive fibrosis in the kidney causes hypoxia and hinders the repair process [41]. To our knowledge, this is the first study that investigated hypoxia and TGF-β1-induced PMT as well as the antifibrotic and antioxidant effects of paricalcitol in pericytes. This study is expected to provide a basis for future research which proves the therapeutic effect of paricalcitol for treating kidney injuries to inhibit AKI–to–CKD transition. However, this study has several limitations. First, we did not evaluate the antifibrotic and antioxidant effects of paricalcitol in the coculture experiment. Since the pericytes are located close to the capillary endothelial cells, the factors produced by the injured endothelial cells can affect the effects of paricalcitol [2]. Second, the detailed mechanism of the protective effect of paricalcitol and how it inhibits the phosphorylation of Smad2 was not evaluated. Further studies are needed to confirm the exact mechanism of action.

In conclusion, hypoxia or TGF-β1 induced PMT in an HIF-1α-dependent manner through the Smad2 signaling pathway. Paricalcitol improved the upregulated PMT via the downregulation of the HIF-1α-dependent pathway and inhibition of the TGF-β1/Smad2 signaling pathway. The pericytes exposed to hypoxia also induced oxidative stress injuries, which were improved due to paricalcitol treatment by increasing the antioxidative enzymes in an HIF-1α-independent manner. Furthermore, our results suggest that paricalcitol could be a potential therapeutic option in the treatment of renal fibrosis by targeting the pericyte biology in hypoxia-induced and TGF-β1-induced injury.

## 4. Materials and Methods

### 4.1. Isolation and Culture of Mouse Kidney Pericytes

The C57BL/6 mouse kidneys were decapsulated, diced, and then incubated at 37 °C for 45 min with Liberase (0.5 mg/mL, Roche, Mannheim, Germany) and DNase (100 U/mL, New England Biolabs, Hitchin, UK) in DMEM/F12 at 37 °C in a shaking water bath. After centrifugation, the cells were resuspended in RBC lysis buffer at 4 °C for 5 min, washed, resuspended in 0.5% BSA buffer containing EDTA, and filtered through a 70- and 40-mm cell strainer. After centrifugation, the pellet was resuspended in 45% (*v*/*v*) Percoll solution and centrifuged at 5525× *g* for 30 min at 4 °C (without braking). The cell layer was then collected from the top layer of the Percoll solution. After centrifugation, the cell pellets were then magnetically labeled with CD146 (LSEC) MicroBeads and separated in accordance with the manufacturer’s instructions. The collected cells were then cultured in a pericyte medium supplemented with 2% FBS, 1% PGS, and 1% P/S (ScienCell, Carlsbad, CA, USA) at 37 °C in 5% CO_2_ and 95% air condition. The primary cultured cells used in the present study were between Passage 2 and 6. The growth medium was changed every 3–4 days.

We confirmed the cultured pericytes via flow cytometry and pericyte-specific marker staining, such as PDGFRβ, CD146, and neuron–glial antigen 2 (NG2) (Supplemental Figure S2). In the fluorescence-activated cell sorting analysis, the cultured pericytes showed an increased population of PDGFRβ, CD146, and NG2 positive cells, and the CD31 positive cell population was not altered. In the immunofluorescence staining analysis, the cultured pericytes also coexpressed CD146/PDGFRβ, CD146/NG2, and PDGFRβ/NG2. The pericyte viability was evaluated using the Cell Counting Kit-8 (CCK-8) assay; pericyte viability was not altered after treatment with paricalcitol (20 ng/mL) and/or TGF-β1 (5 ng/mL) for 24 h.

### 4.2. Cell Treatments

After growth factor starvation for 24 h, the pericytes were pretreated with or without paricalcitol (20 ng/mL) for 90 min and subsequently stimulated by TGF-β1 (5 ng/mL; R&D systems, Minneapolis, MN, USA) for 48 h. For hypoxia, the pericytes were cultured in 1% oxygen and 5% CO_2_ in the hypoxia workstation (Invivo2 400, Baker Ruskinn, Sanford, ME, USA).

### 4.3. Immunofluorescence Staining of Pericytes

The pericytes were cultured on chamber slides and fixed with 3.7% paraformaldehyde in PBS for 30 min. Moreover, the pericytes were washed with PBS and permeabilized with 0.3% Triton for 15 min. The fixed cells were incubated with primary antibodies to PDGFRβ (1:50), CD146 (1:50), and NG2 (1:50) overnight at 4 °C. After rewashing the pericytes, they were incubated with the secondary antibodies (Alexa Fluor 488 and Alexa Fluor 594, Life Technologies, Eugene, OR, USA) for 2 h at room temperature. The nucleus was counterstained with 4′,6-diamidino-2-phenylindole (DAPI), and the slides were mounted with an antifade mounting reagent (Molecular Probes). The sections were examined by a Zeiss confocal scanning laser microscope using the LSM 5 Exciter (Carl Zeiss, Oberkochen, Germany).

### 4.4. Cell Viability Assay

The pericytes were seeded in 96-well plates and incubated using a serum-free medium for 24 h. After starvation, the pericytes were treated with paricalcitol (20 ng/mL) and/or TGF-β1 (5 ng/mL). The cell viability was analyzed using the CCK-8 assay (Dojindo Laboratories, Kumamoto, Japan) in accordance with the manufacturer’s instructions. The absorbance was measured at 450 nm using a microplate (SPARK 10M, Tecan, Durham, NC, USA). The value was expressed as percentage of control.

### 4.5. Scratch Cell Migration Assay

The pericytes were plated into 96-well IncuCyte ImageLock Plates (Essen BioScience, AnnArbor, MI, USA) and incubated overnight. The medium was changed to complete medium with mitomycin-C (10 μg/mL, M4287; MilliporeSigma, Burlington, MA, USA) for 2 h to inhibit cell proliferation. Each cell monolayer was scratched using a 96-pin WoundMaker (IncuCyte Zoom Live-Cell Imaging System; Essen BioScience) in accordance with the manufacturer’s instructions. After scratching, to study the effect of paricalcitol on cell migration, the pericytes were pretreated with paricalcitol (20 ng/mL) for 90 min and subsequently incubated with TGF-β1 (5 ng/mL) for 48 h. After treatment, the images were obtained, saved, and registered in the IncuCyte software system every 4 h. The data were analyzed using an integrated metric, and the values were expressed as relative wound density.

### 4.6. Transwell Cell Migration Assay

The pericytes were treated with TGF-β1 (5 ng/mL) for 24 h after preincubation with paricalcitol (20 ng/mL) for 90 min. Moreover, the cells were detached with trypsin–EDTA and plated on 24-well Transwell inserts (1 × 10^4^ cells/well, 8 mm pore size; 6.5 mm Transwell [#3422], Corning, NY, USA). After 24 h, the cells that had migrated to the lower face of the inserts were fixed with 3.7% paraformaldehyde for 10 min. On the other hand, the migrated cells on the lower face were stained with 0.1% crystal violet (V5265; Sigma–Aldrich, St. Louis, MO, USA) for 5 min, and the cells in 10 randomly selected fields were counted under a light microscope at ×100 magnification.

### 4.7. Transfection of Pericytes with HIF-1α siRNA

Mouse HIF-1α siRNA and nontargeting (negative control) siRNA were purchased from Dharmacon (Chicago, IL, USA). The transfection was performed using Lipofectamine RNAiMax (Invitrogen, Paisely, UK), in accordance with the manufacturer’s instructions. The pericyte cells (5.0 × 10^4^) were seeded 1 day prior to transfection and cultured to reach 40–50% confluence on the following day. The RNAi duplexes for HIF-1α were mixed with Lipofectamine to form a transfection complex that was added to the plated cells. After 6 h of incubation, the cells were pretreated with paricalcitol (20 ng/mL) for 90 min, then transferred into a hypoxia chamber and exposed hypoxia for 24 h. The transfected cells were used for quantitative realtime reverse transcription-polymerase chain reaction (qRT-PCR).

### 4.8. RNA Extraction and Quantitative Realtime PCR Analysis

The total RNA was extracted from Trizol (Invitrogen, Carlsbad, CA, USA)-treated pericytes in accordance with the manufacturer’s instructions. One microgram of total RNA was transcribed reversely by using the PrimeScript cDNA Synthesis kit (Takara Shuzo Co., Otsu, Japan). All primers for realtime PCR (Supplemental Table S1) were designed using the Primer Express V1.5 software (Applied Biosystems, Foster City, CA, USA). Quantitative realtime PCR was performed on the ABI PRISM 7500 Sequence Detection System (Applied Biosystems, Foster City, CA, USA) using the SYBR Green PCR Master Mix (Applied Biosystems, Foster City, CA, USA). All of the samples were calculated using the comparative Ct method for the relative quantification of gene expression and normalization with regard to the GAPDH expression.

### 4.9. Western Blot Analysis

After treatment, the total proteins (25 μg) were separated via 10% sodium dodecyl sulfate–polyacrylamide gel electrophoresis and transferred to a nitrocellulose membrane. The membrane was blocked with 10% skim milk for 1h at room temperature and incubated overnight at 4 °C with primary antibodies against phospho-Smad2 (1:1000, Millipore), Smad2 (1:200, Invitrogen), phospho-Smad3 (1:1000, Cell Signaling, Danvers, MA, USA), Smad3 (1:1000, Cell Signaling), TGF-β1 (1:1000, Santa Cruz, Dallas, TX, USA), and GAPDH (1:2000, Cell Signaling). After washing, the membrane was incubated with horseradish peroxidase-conjugated secondary antibodies (P0448, Dako, Glostup, Denmark) for 1 h. The membrane was detected using ECL reagents (Amersham Bioscience, Piscataway, NJ, USA) and visualized on an ImageQuant™ LAS 4000 system (GE Healthcare Life Sciences, Tokyo, Japan). The protein band densities were quantified using the Scion Image software (Scion, Frederick, MD, USA).

### 4.10. Statistical Analysis

The data are presented as means ± standard errors and derived from at least three independent experiments. The statistical analyses were performed using the nonparametric Kruskal–Wallis test, followed by a posthoc analysis using the Mann–Whitney *U* test with Bonferroni correction. The analysis was performed using SPSS (version 20.0, IBM Corp., Armonk, NY, USA). A *p*-value of less than 0.05 was considered statistically significant.

## Data Availability

The data presented in this study are available on request from the corresponding author.

## Supplementary Materials

The following are available online at https://www.mdpi.com/article/10.3390/ijms22189751/s1.
