# Supplementary material for: Paricalcitol Improves Hypoxia-Induced and TGF-β1-Induced Injury in Kidney Pericytes
Source: Int J Mol Sci. 2021 Sep 9;22(18):9751. doi: 10.3390/ijms22189751 (PMC8472327; doi:10.3390/ijms22189751)

Paricalcitol improves hypoxia-induced and TGF-β1–induced injury in kidney pericytes

Jeong-Hoon Lim, Ju-Min Yook, Se-Hyun Oh, Soojee Jeon, Hee Won Noh, Hee-Yeon Jung, Ji-Young Choi, Jang-Hee Cho, Chan-Duck Kim, Yong-Lim Kim, and Sun-Hee Park

Department of Internal Medicine, School of Medicine, Kyungpook National University, Kyungpook National University Hospital, Daegu, South Korea

Supplementary Information Listing:

Supplemental Table S1, Figure S1, and Figure S2

*Corresponding author: Sun-Hee Park, MD, PhD

Professor

Division of Nephrology, Department of Internal Medicine

Kyungpook National University Hospital, 130 Dongdeok-ro, Jung-gu, Daegu, 41944, South Korea

Tel: +82-53-200-5547

Fax: +82-53-426-9464

Email: sh-park@knu.ac.kr

Supplemental Table S1. Primer sequences used in quantitative real-time PCR

Target	Primer	Sequences
mouse α-SMA	Forward	5'-CTGACAGAGGCACCACTGAA-3'
	Reverse	5'-CATCTCCAGAGTCCAGCACA-3'
mouse Fibronectin	Forward	5'-ACCACCGGCCACAACTACAA-3'
	Reverse	5'-TCTAACGGCATGAAGCACTCA-3'
mouse Vimentin	Forward	5'-GCTGCAGGCCCAGATTCA-3'
	Reverse	5'-TTCATACTGCTGGCGCACAT-3'
mouse MMP-9	Forward	5'-CCAGACGTGGGTCGATTCC-3'
	Reverse	5'-TGTCTCGCGGCAAGTCTTC-3'
mouse PDGFRβ	Forward	5'-CGTGTCCCTGCCTCTTTCC-3'
	Reverse	5'-GAAGACACAGAGTGGAGGTAGAGAAAT-3'
mouse TGF-β1	Forward	5'-TTCCTAGCCAACTTCTGCCA-3'
	Reverse	5'-ATCCACCACCATGTCTTCGT-3'
mouse HIF-1α	Forward	5'- GCGGGCACCGATTCG-3'
	Reverse	5'-TTCAGAACTCATCTTTTTCTTCTCGTT-3'
mouse PHD3	Forward	5'-TCGCTTCCTCCCGAACTCT-3'
	Reverse	5'- CAGAAACGAGGGTGGCTAACTT-3'
mouse GLUT-1	Forward	5'-CTGGGCAAGTCCTTTGAGATG-3'
	Reverse	5'-CCGCAGTACACACCGATGAT-3'
mouse SOD1	Forward	5'-CAAAGGTGGAAATGAAGAAAGTACAA-3'
	Reverse	5'-GGGAATGTTTACTGCGCAATC-3'
mouse SOD2	Forward	5'-GGTAGGGCCTGTCCGATGAT-3'
	Reverse	5'-GTCCAGTTCTCCAGAGATATACAATTCA-3'
mouse Catalase	Forward	5'-CGACCAGGGCATCAAAAACT-3'
	Reverse	5'-ATTGGCGATGGCATTGAAA-3'
mouse Glutathione peroxidase	Forward	5'-GGAACAACTACCCGGGACTACA-3'
	Reverse	5'-GATGTCCGAACTGGTTGCAA-3'
mouse GAPDH	Forward	5'-TAAAGGGCATCCTGGGCTACACT-3'
	Reverse	5'-TTACTCCTTGGAGGCCATGTAGG-3'

Abbreviations: α-SMA, alpha-smooth muscle actin; MMP-9, matrix metallopeptidase-9; PDGFRβ, platelet derived growth factor receptor beta; HIF, hypoxia-inducible factor; PHD3, prolyl hydroxylase 3; GLUT-1, glucose transporter 1; SOD, superoxide dismutase.

Supplemental Figure S1. The changes of profibrotic markers according to TGF-β1 concentration.

TGF-β1 increased mRNA expression of profibrotic markers in a dose-dependent manner. (A) α-SMA. (B) Fibronectin. (C) Vimentin. (D) MMP-9. The data is presented as means ± standard errors. n = 4 per each group. $^{*}P < 0.05$ vs. control; $^{**}P < 0.01$ vs. control; $^{***}P < 0.001$ vs. control Abbreviations: α-SMA, alpha-smooth muscle actin; MMP-9, matrix metalloproteinase-9.

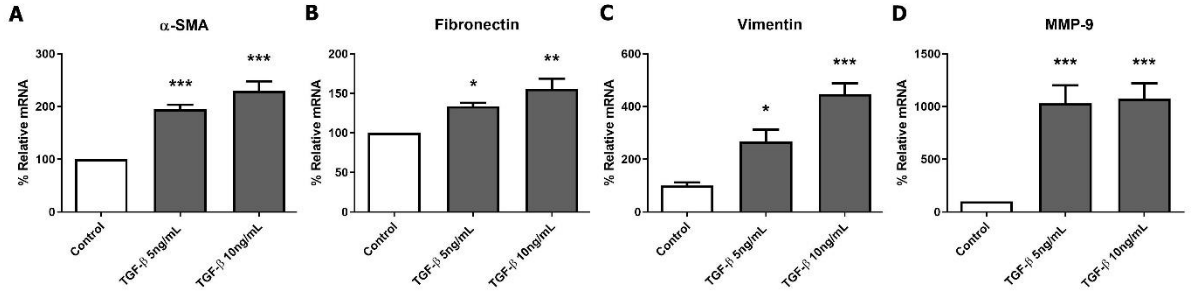

Supplemental Figure S2. Cultured pericytes and cell viability assay.

(A) Cultured pericytes were confirmed by fluorescence-activated cell sorting analysis. (B) Immunofluorescence staining of pericyte markers in cultured pericytes. (C) Pericyte viability after treatment of TGF-β1 with/without paricalcitol in CCK-8 assay.

Abbreviations: CCK-8, Cell Counting Kit-8; C, control group; P20, paricalcitol 20 ng/mL treatment group, T5, TGF-β1 5 ng/mL treatment group; T5+P20, TGF-β1 5 ng/mL and paricalcitol 20 ng/mL cotreatment group.

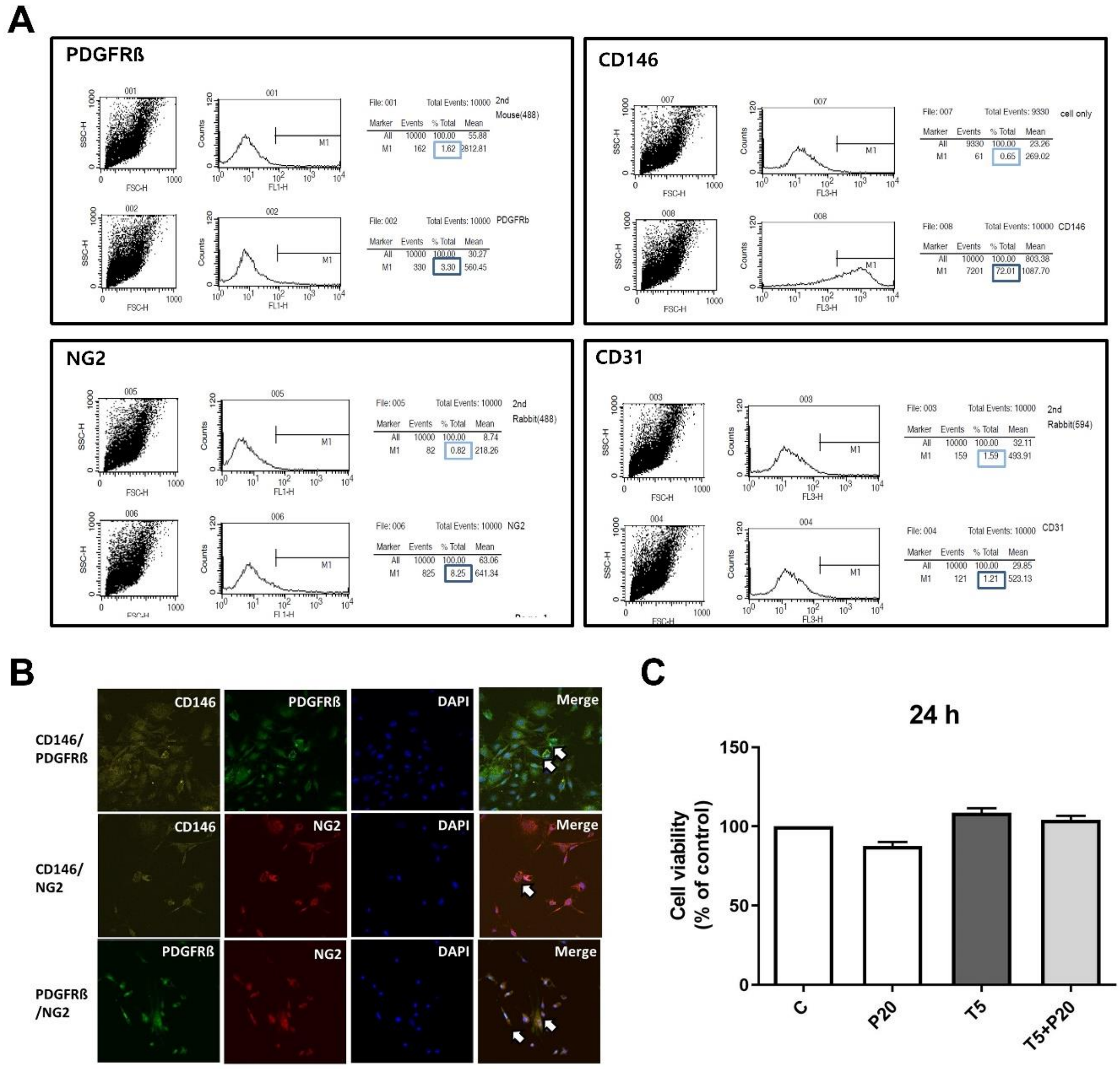

Supplement: Supplementary file 1 [file ijms-22-09751-s001.zip › ijms-1345954-Supplementary.pdf]
